# Cdt1 degradation to prevent DNA re-replication: conserved and non-conserved pathways

**DOI:** 10.1186/1747-1028-2-18

**Published:** 2007-06-12

**Authors:** Youngjo Kim, Edward T Kipreos

**Affiliations:** 1 Department of Cellular Biology, University of Georgia, Athens, GA 30602-2607 USA

## Abstract

In eukaryotes, DNA replication is strictly regulated so that it occurs only once per cell cycle. The mechanisms that prevent excessive DNA replication are focused on preventing replication origins from being reused within the same cell cycle. This regulation involves the temporal separation of the formation of the pre-replicative complex (pre-RC) from the initiation of DNA replication. The replication licensing factors Cdt1 and Cdc6 recruit the presumptive replicative helicase, the Mcm2-7 complex, to replication origins in late M or G1 phase to form pre-RCs. In fission yeast and metazoa, the Cdt1 licensing factor is degraded at the start of S phase by ubiquitin-mediated proteolysis to prevent the reassembly of pre-RCs. In humans, two E3 complexes, CUL4-DDB1^CDT2 ^and SCF^Skp2^, are redundantly required for Cdt1 degradation. The two E3 complexes use distinct mechanisms to target Cdt1 ubiquitination. Current data suggests that CUL4-DDB1^CDT2^-mediated degradation of Cdt1 is S-phase specific, while SCF^Skp2^-mediated Cdt1 degradation occurs throughout the cell cycle. The degradation of Cdt1 by the CUL4-DDB1^CDT2 ^E3 complex is an evolutionarily ancient pathway that is active in fungi and metazoa. In contrast, SCF^Skp2^-mediated Cdt1 degradation appears to have arisen relatively recently. A role for Skp2 in Cdt1 degradation has only been demonstrated in humans, and the pathway is not conserved in yeast, invertebrates, or even among other vertebrates.

## Cdt1 degradation and the control of DNA replication

To maintain genome integrity, DNA replication must be strictly regulated to occur only once per cell cycle. Replication is, therefore, tightly regulated to prevent the re-initiation of DNA replication within the same S phase. A failure to restrict DNA replication results in 're-replication', in which the genome is over-replicated within the same cell cycle via origin re-firing. In eukaryotes, the extent of DNA replication is controlled by temporally restricting the assembly of the pre-replicative complex (pre-RC) through a process termed 'replication licensing' (reviewed in [1,2]). Pre-RCs form on replication origins through the sequential binding of DNA replication proteins during late mitosis or G1 phase. The six-member origin recognition complex (ORC) binds replication origins on newly-synthesized chromatin. During late mitosis or G1 phase, the replication licensing factors Cdt1 and Cdc6 are recruited to the origin. Cdt1 and Cdc6 together load the presumptive replicative helicase, the Mcm2-7 complex, onto the origin to complete pre-RC formation. During S phase, pre-RCs are activated by phosphorylation via CDK and DDK (Dbf4-dependent kinase) activity. This phosphorylation allows the recruitment of essential replication factors, including Cdc45, Mcm10, RPA, proliferating cell nuclear antigen (PCNA), and DNA polymerases α and δ.

Cdt1 and Cdc6 are essential loading factors for the Mcm2-7 complex, and they are negatively regulated during S phase to ensure that the Mcm2-7 complex cannot re-bind to origins that have already fired. In budding yeast, Cdt1 is exported from the nucleus during S phase [3]. In contrast, fission yeast and metazoan Cdt1 homologs are degraded during S phase [1,2,4]. The other replication licensing factor, Cdc6, is regulated by degradation during S phase in budding and fission yeast, while in metazoa, Cdc6 is exported from the nucleus [1,2,4,5]. In most eukaryotic species examined, redundant regulation prevents reassembly of pre-RCs in S phase. The exact regulation varies between eukaryotes, and can include controls of each of the pre-RC components: Cdt1, Cdc6, ORC subunits, and the Mcm2-7 complex [1,2,4]. In all eukaryotic species examined, Cdt1 is a major focus of replication licensing regulation.

In *Drosophila *and vertebrates, Cdt1 activity is redundantly regulated by its degradation and the binding of a Cdt1-inhibitor called Geminin [2]. Loss of Geminin leads to re-replication in *Drosophila *and in certain human cell lines but not in others [6-11]. In human HeLa cells, Cdt1 is degraded prior to the expression of Geminin, suggesting that Geminin is a back-up system that functions after the majority of Cdt1 has been degraded [12]. HeLa cells do not undergo re-replication when Cdt1 degradation is blocked or when Geminin is inactivated; however, when both pathways are deregulated simultaneously, re-replication is observed, indicating that the two pathways redundantly restrain Cdt1 activity [13].

In both *C. elegans *and *Xenopus *embryos, loss of Geminin is not associated with re-replication [14-16]. In contrast, Cdt1 degradation is more critical for regulating DNA replication in these species. A failure to degrade CDT-1 in *C. elegans *is associated with re-replication [17]. Likewise, the expression of a non-degradable Cdt1 (but not wild-type Cdt1) induces re-replication in *Xenopus *egg extract [18].

Cdt1 is degraded by the ubiquitin-proteasome system. In this pathway, ubiquitin ligases (E3s) provide the specificity for the degradation because they bind specific substrates and then facilitate the transfer of ubiquitin from the ubiquitin conjugating enzyme (E2) to the substrate [19]. The covalent attachment of a tandem array of ubiquitins to the substrate (in the proper linkage) induces the degradation of the substrate by the 26S proteasome [20].

In humans, two distinct E3 complexes, CUL4-DDB1^CDT2 ^and SCF^Skp2^, have been reported to target Cdt1 for ubiquitin-mediated degradation. Both of these E3s are members of the cullin-RING ligase (CRL) class of ubiquitin ligases. The two E3 complexes utilize distinct mechanisms for targeting Cdt1 ubiquitination. In this review, we will focus on the regulation of Cdt1 degradation in different species and explore the conservation of pathway components and mechanisms across species and phyla.

## The CUL4-DDB1 complex targets Cdt1 for degradation

Studies in *C. elegans *first suggested the involvement of CUL4 in Cdt1 degradation. The inactivation of the *C. elegans cul-4 *gene by RNAi causes proliferating cells to arrest in S phase and undergo massive levels of DNA re-replication [17]. The DNA content of the re-replicating cells increases up to 100 C (where 2 C is the normal diploid DNA content). In *C. elegans*, as in vertebrates and fission yeast, CDT-1 is degraded as cells enter S phase [17]. However, when *cul-4 *is inactivated, CDT-1 is not degraded in S phase, but instead accumulates in the re-replicating cells [17]. Reduction of CDT-1 levels by half abolishes the re-replication in *cul-4 *RNAi cells, indicating that CDT-1 accumulation is a critical factor in causing the re-replication. This work showed that CUL-4 negatively regulates CDT-1 levels, but did not address whether CDT-1 is a direct target of the CUL-4 complex. It was subsequently shown in humans, *Xenopus*, fission yeast, and *C. elegans *that the CUL4 ubiquitin ligase directly mediates Cdt1 degradation during S phase [13,16,21,22].

In humans and *Drosophila*, Cdt1 is rapidly degraded in response to DNA damage induced by UV- or γ-irradiation, presumably to prevent DNA replication until the DNA damage can be repaired [23]. CUL4 is also required for this Cdt1 degradation pathway [23,24]. The CUL4-mediated degradation of Cdt1 upon DNA damage occurs independently of DNA replication or the classic DNA damage pathway that includes the ATM/ATR and CHK1/CHK2 kinases [23]. The CUL4-dependent Cdt1 degradation in response to DNA damage can occur throughout the cell cycle (in G1, S, and G2 phases) [13,22-24]. Given the cell cycle-independent nature of the degradation, it is fair to ask whether the degradation is simply to prevent DNA replication in S phase or whether there is an additional cell cycle-independent role.

## The modular structure of CUL4-DDB1 ubiquitin ligase complex

**Figure 1:**
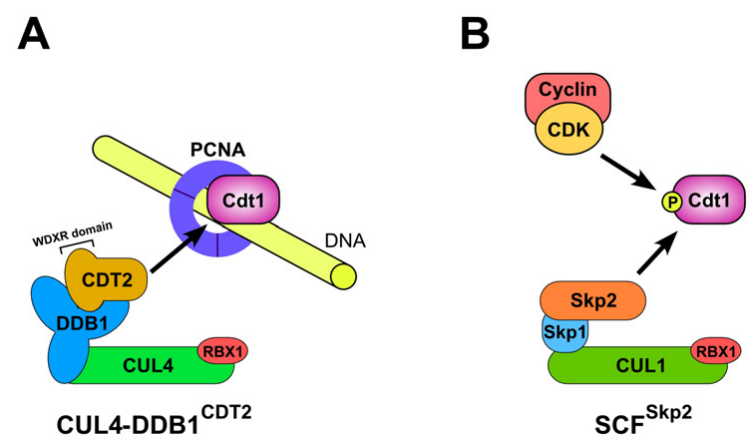
**Two distinct molecular pathways for Cdt1 degradation**. (A and B) CUL4-DDB1^CDT2 ^and SCF^Skp2 ^CRL ubiquitin ligase complexes have similar modular structures: a cullin; a common RING H2-finger protein Rbx1; an adaptor protein, DDB1 or Skp1; and an SRS, CDT2 or Skp2. In the CUL4-DDB1^CDT2 ^complex, CDT2 binds to DDB1 through a WD-repeat region with a specific signature, termed a 'WDXR' or 'DXR' domain (marked in figure). In the SCF^Skp2 ^complex, Skp2 binds to Skp1 through an F-box motif (not marked). (A) CUL4-DDB1^CDT2 ^targets Cdt1 for degradation after Cdt1 binds to PCNA on chromatin. CDT2 is proposed to directly bind Cdt1 after Cdt1 binds to PCNA, although the CDT2-Cdt1 interaction has not yet been formally demonstrated. (B) SCF^Skp2 ^targets Cdt1 for degradation after CDK/Cyclin complexes phosphorylate Cdt1. See text for details.

The cullin-RING ubiquitin ligase (CRL) complexes represent the largest super-family of multisubunit E3s in eukaryotes [25]. The prototype of the CRL is the SCF complex, which comprises: the cullin CUL1 (which forms a rigid scaffold); the RING-H2 finger protein Roc1/Rbx1/Hrt1 (which is bound to the C-terminus of CUL1 and facilitates ubiquitin conjugating enzyme loading and activation); the adaptor protein Skp1 (which is bound to the N-terminus of the cullin); and a variable F-box protein (which is linked to Skp1 through the F-box motif and is the substrate-recognition subunit, SRS) (Fig. 1B). Other cullins form similar CRL complexes that contain the same RING finger protein but have different adaptors and SRSs.

CUL4 complexes contain the RING finger protein Rbx1 and the adaptor DDB1 (damaged DNA-binding protein) [25] (Fig. 1A). DDB1 appears to be an integral component of all CUL4-dependent processes; and loss of DDB1 provides the same phenotypes and molecular defects as loss of CUL4 in *C. elegans*, humans, and fission yeast [13,16,22,26].

The crystal structure of the CUL4-DDB1 E3 complex has recently been determined [27,28]. DDB1 is a large multi-domain protein that contains three β-propeller domains. One β-propeller domain binds to the N-terminus of the rod-shaped CUL4, while the other two β-propeller domains form a bi-lobed domain that has multiple potential interaction sites for protein binding [27,28] (Fig. 1A). A number of proteins have been identified as DDB1 interactors that are presumed or known to function in substrate binding: hDET/hCOP1; CDT2/L2DTL/DTL; DDB2; CSA; and paramyxovirus V proteins [28-32]. Additionally, four groups have used biochemical and proteomic approaches to identify a novel family of WD40-repeat proteins that bind to the CUL4-DDB1 complex [27,33-35]. The majority of these WD-repeat proteins possess a variant WD-repeat sequence that is referred to as a 'WDXR', or 'DXR', and which mediates binding to DDB1 [27,33-35] (Fig. 1A). This WD repeat protein family has been referred to by three names: DCAF (DDB1- and CUL4-associated factors); CDW (CUL4 and DDB1-associated WD40 repeat proteins); and DWD (DDB1-binding and WD40 repeat proteins) [27,33-35].

The DCAF family is predicted to contain ~90 members in mammals, of which at least 49 have been shown to physically interact with CUL4 or DDB1, mainly by co-expression/co-IP in mammalian cells [27,33-35]. Five members of the DCAF family are known to function as SRSs, and the functions of their respective complexes are as follows. CUL4-DDB1^CSA ^targets the degradation of the nucleotide excision repair protein CSB [36]. CUL4-DDB1^hDET1-hCOP1^, which includes a dimeric SRS consisting of hDET1 and hCOP1, targets the degradation of the transcription factor c-jun [31]. CUL4-DDB1^VprBp ^is known to be hijacked by the Vpr protein of the human immunodeficiency virus (HIV) to induce cell cycle arrest [37]. CUL4-DDB1^DDB2 ^mediates stable ubiquitin modifications of histones H2A, H3 and H4 and the nucleotide excision repair protein XPC [38-40]. Finally, CUL4-DDB1^CDT2 ^has been implicated in the degradation of mammalian p53, fission yeast Spd1, and Cdt1 (described below), [22,26,29,33,41,42]. It is likely that there will be additional CUL4-DDB1 complexes containing different DCAF proteins that function in a wide-range of molecular and physiological processes.

## CDT2 and PCNA are required for Cdt1 degradation

The DCAF protein CDT2 physically interacts with CUL4 and DDB1 *in vivo *[26,33,41]. In humans, *Xenopus*, zebrafish, and fission yeast, CDT2 is required for Cdt1 degradation during S phase and in response to DNA damage [22,26,33,41]. In *Xenopus *egg extract, CDT2 is required to load the CUL4-DDB1^CDT2 ^complex onto chromatin in response to Cdt1 binding to PCNA, suggesting that the interaction of CDT2 with Cdt1 mediates the localization [33] (Fig. 1A). These findings suggest that CDT2 is the SRS for the complex. However, this has not yet been formally established, as there are no reports that CDT2 can bind directly to Cdt1. There is also an intriguing finding that inactivating human CDT2 reduces the association of DDB1 with CUL4, suggesting that CDT2 may have an additional or alternative function to regulate CUL4-DDB1 complex formation [26].

There is also evidence that DDB1 may function directly as the SRS for Cdt1 binding. It has been reported that purified human Cdt1 can bind directly to purified DDB1 [24]. Further, *in vitro *translated *C. elegans *DDB-1 made with a wheat germ extract binds to bacterially-produced recombinant GST-CDT-1 [16]. The *C. elegans *experiments, however, do not rule out the possibility that a plant protein from the wheat germ extract bridges the interaction between DDB-1 and CDT-1. Additional experiments will be required to clarify exactly how the CUL4-DDB1^CDT2 ^complex binds to Cdt1.

In *Xenopus *egg extract, the degradation of Cdt1 by CUL4-DDB1^CDT2 ^in S phase requires the interaction between Cdt1 and PCNA [18,33] (Fig. 1A). Cdt1 binds PCNA through a PCNA-interacting protein (PIP) box motif in the Cdt1 N-terminus [18]. The PIP box is also required for the CUL4-DDB1-mediated degradation of Cdt1 during S phase in humans and *C. elegans *[13,16,43]. The degradation of Cdt1 in response to UV irradiation has similarly been shown to require the association of Cdt1 with PCNA in humans, *Drosophila*, and fission yeast [13,18,26,43,44]. This suggests that the two distinct Cdt1 degradation events, occurring in response to DNA damage or S-phase entry, are triggered by the same molecular signal: Cdt1 binding to chromatin-associated PCNA. PCNA forms a trimeric ring structure that is loaded onto DNA during both DNA replication and DNA repair [45,46]. One can hypothesize that chromatin-loaded PCNA (potentially in conjunction with other factors) is sufficient to promote Cdt1 binding and its subsequent degradation.

## SCF^Skp2 ^functions redundantly with CUL4-DDB1^CDT2 ^to degrade Cdt1 in humans

In humans, the SCF^Skp2 ^E3 complex also targets Cdt1 for degradation. Human Cdt1 is phosphorylated by cyclin-CDK complexes, and the phosphorylation is dependent on a cyclin-binding (Cy) motif within Cdt1 [47,48]. The phosphorylation on threonine 29 within the N-terminus of Cdt1 is required for its interaction with Skp2 [49]. Mutating the N-terminal CDK-phosphorylation sites of Cdt1 increases its half-life in asynchronous human cells [47]. Similarly, siRNA depletion of Skp2 increases the level of Cdt1 in asynchronous human cells [13,50]. These results indicate that SCF^Skp2 ^regulates Cdt1 levels in response to CDK-phosphorylation (Fig. 1B).

There have been differing reports on the effect of inactivating the SCF^Skp2 ^pathway on Cdt1 levels in human S-phase cells. One study indicated that Skp2 was required to allow S-phase degradation of Cdt1 [50]. A second study indicated that mutation of the cyclin-binding motif of Cdt1 (which prevents Skp2 binding) does not block the majority of Cdt1 degradation in S phase, although higher residual levels of Cdt1 protein are observed in S-phase cells [48]. Finally, three other reports indicate that inactivation of the SCF^Skp2^-mediated Cdt1 degradation pathway does not stabilize Cdt1 during S phase [13,43,49]. Recent work has clarified these apparent contradictions by showing that in human cells both SCF^Skp2 ^and CUL4-DDB1^CDT2 ^pathways redundantly target Cdt1 for degradation during S phase [13,43].

Analysis of published results suggests that SCF^Skp2 ^mediates Cdt1 degradation throughout the cell cycle. This conclusion is based on the observation that Skp2 siRNA depletion in asynchronous cells leads to a three-fold increase in Cdt1 levels, even though Skp2 siRNA treatment does not affect S- or G2-phase levels of Cdt1 (because Cdt1 is still targeted for degradation by CUL4-DDB1^CDT2^) [13]. This implies that Cdt1 levels must increase in non-S- or G2-phase cells upon Skp2 siRNA treatment (presumably G1 phase cells). As described above, Skp2 redundantly targets Cdt1 for degradation during S and G2 phases. Therefore, it can be concluded that Skp2 targets Cdt1 degradation throughout the cell cycle. In contrast, CUL4-DDB1-mediated Cdt1 degradation is S-phase specific [13].

## Cdt1 degradation in other metazoa and yeast

The roles of Skp2 and CUL4 in degrading Cdt1 have also been explicitly compared in *C. elegans*. Inactivation of *C. elegans cul-4 *or *ddb-1 *fully stabilizes CDT-1 during S phase [16,17]. In contrast, the *C. elegans *Skp2 homolog, *skpt-1*, does not contribute to CDT-1 degradation or re-replication even in a sensitized *ddb-1 *mutant background [16]. *skpt-1 *null mutant homozygotes are completely viable and appear overtly wild-type with the exception of a low-penetrance gonad migration defect, indicating that the gene is not required for any essential functions [16].

In *Xenopus *egg extract, CDK-phosphorylation of Cdt1 is not required for Cdt1 degradation [21]. This implies that SCF^Skp2 ^is not required for Cdt1 degradation because CDK-phosphorylation of Cdt1 is a prerequisite for recognition by human Skp2 [47,48], and most SCF^Skp2 ^substrates must be phosphorylated to be recognized [25]. In contrast, CUL4-DDB1 is essential for Cdt1 degradation in *Xenopus *egg extract, with DDB1 depletion blocking Cdt1 degradation during S phase [18]. These results suggest that CUL4-DDB1^CDT2 ^is the predominant E3 for Cdt1 degradation in *Xenopus*, and that SCF^Skp2 ^either has no role or has only a minor, subservient role in Cdt1 degradation.

In *Drosophila*, mutation of all of the N-terminal CDK-phosphorylation sites of Cdt1 is not able to block S-phase degradation, although it does provide a limited increase in overall stability [51]. This indicates that a phosphorylation-dependent pathway (and by implication SCF^Skp2^) either is not involved or is redundant for Cdt1 degradation during S phase in *Drosophila*. There are currently no reports on the function of the fly Skp2 homolog.

Fission yeast does not have a recognizable Skp2 homolog, but does express the CUL4-DDB1^CDT2 ^complex. Fission yeast CUL4-DDB1^CDT2 ^is essential for the degradation of Cdt1 during S phase and in response to DNA damage, indicating that it is the dominant pathway for regulating Cdt1 levels [22]. Taken together, these studies suggest that SCF^Skp2^-mediated degradation of Cdt1 is not conserved in non-mammalian species (Table 1).

**Table 1:** Cdt1 degradation directed by CUL4-DDB1^CDT2 ^and SCF^Skp2 ^in different species.

	**Degradation of Cdt1 by**	
	
**Species**	**CUL4-DDB1**^**CDT2**^	**SCF**^**Skp2**^
Human	Yes	Yes
Mice	Yes	(No?)^a^
Frogs	Yes	No
Flies	Yes	?^b^
Nematodes	Yes	No
Fission yeast	Yes	No^c^

^a ^Available evidence suggests that SCF^Skp2 ^does not direct Cdt1 degradation in mice, however direct experiments have not been performed (see text).

^b ^No published studies have addressed the role of Skp2 in *Drosophila*.

^c ^Fission yeast lack a recognizable *Skp2 *homolog.

See text for references and discussion.

## Is the SCF^Skp2^-dependent Cdt1 degradation pathway conserved in mice?

The studies described above suggest that SCF^Skp2^-mediated degradation of Cdt1 is not conserved in yeast, invertebrates, or even the vertebrate *Xenopus laevis*. It is therefore valid to ask whether SCF^Skp2^-mediated Cdt1 degradation is conserved among mammals; and in fact, there is evidence that casts doubt on the conservation of the pathway in mice. Inactivation of Skp2 by siRNA treatment in human cells leads to an approximately three-fold increase in the steady state level of Cdt1 [13,50]. However, Skp2^-/- ^knockout mice or Skp2^-/- ^MEFs (mouse embryonic fibroblasts) do not have elevated levels of Cdt1 [13,52]. In contrast, DDB1^-/- ^knockout mice have elevated Cdt1 levels in proliferating tissues [53]. Further, Cdt1 protein level is stabilized after UV-irradiation in DDB1^-/- ^MEFs [53]. These results indicate that in mice, the CUL4-DDB1 complex is required non-redundantly for proper Cdt1 degradation during normal cell cycle progression and in response to DNA damage; in contrast, loss of Skp2 does not perturb these processes.

It is interesting that Skp2^-/- ^knock-out mice are completely viable and fertile [54]. This is particularly striking in light of the long list of human Skp2 substrates, including important cell cycle and transcriptional regulators: Cdt1, Orc1, p27^Kip1^, p21^Cip1^, cyclin E, cyclin D, cyclin A, c-Myc, b-Myb, p130/pRb2, E2F-1, p57^Kip2^, MKP-1, RAG-2, FOXO1, and Cdk9 [47,48,50,54-73]. Although Skp2^-/- ^mice are viable, they exhibit a minor defect of polyploidy and extra centrosomes in the cells of a few tissues [54]. Both of these defects arise as secondary consequences of a failure of these cells to enter mitosis, with the affected cells subsequently re-entering the next cell cycle and duplicating their DNA and centrosomes [52]. Significantly, the mitotic defect is suppressed by co-inactivation of p27^Kip1^, suggesting that the inability to degrade p27^Kip1 ^causes the defect [52]. The lack of phenotypes associated with a failure to degrade other potential substrates suggests either that they are not substrates in mice, that their degradation is not important for development, or that they are under redundant control with other degradation pathways. Taken together, the available evidence suggests that CUL4-DDB1^CDT2 ^is the predominant ubiquitin ligase to mediate Cdt1 degradation in mice, and that SCF^Skp2 ^either does not target Cdt1 for degradation or does so only as a minor pathway that cannot compensate for loss of CUL4-DDB1^CDT2^.

## When did genes for the two Cdt1-degradation pathways arise during evolution?

**Figure 2:**
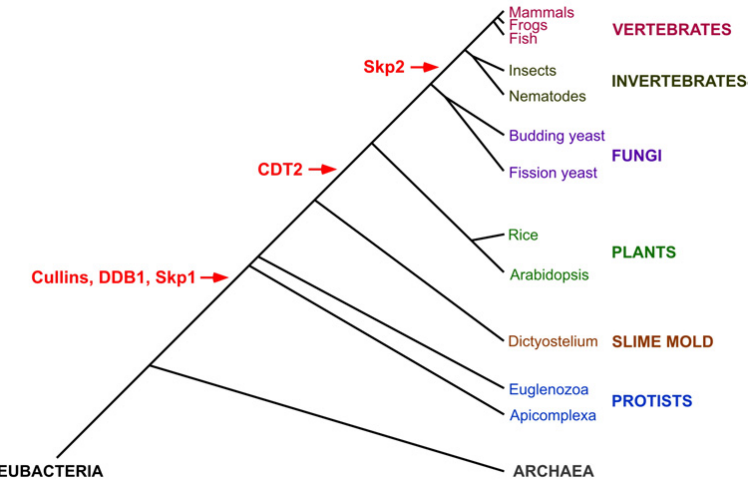
**The genesis of CUL4-DDB1^**CDT2 **^and SCF^**Skp2 **^E3 components**. CUL4-DDB^CDT2 ^and SCF^Skp2 ^complex components were examined in representative organisms of diverse phyla (Table 2). A phylogenetic tree of the taxa analyzed, from eubacteria to mammals, is presented. Note that distances between branches are not to scale. Species and major classifications are color-coordinated, and the temporal locations of the presumed origins of E3 component genes are in red. CUL1-like and CUL4-like cullins, as well as their adaptor proteins DDB1 and Skp1, respectively, appear to have arisen early in eukaryotes, as they are absent from archaea and bacteria but are found in the eukaryotes examined. CDT2, the SRS for a CUL4-DDB1 E3 complex, appears to have arisen prior to the genesis of green plants. Skp2, the SRS for a CUL1 E3 complex, appears to have arisen after the genesis of fungi but prior to the genesis of metazoa. The branching order is based on a phylogenetic analysis using rRNA [76]. Note that other phylogenies, based on protein sequences, reverse the order of plants and slime molds [77]. Combining our genomic data with this alternative branching of phyla (not shown) would imply that CDT2 was created prior to plants in the main eukaryotic lineage but then lost within the slime mold lineage.

To determine when the genes for the CUL4-DDB1^CDT2 ^and SCF^Skp2 ^complexes arose during evolution, we analyzed divergent species using reciprocal BLAST searches [74]. We limited our analysis to those organisms in which the whole genome had been sequenced, so that a failure to detect a gene would be meaningful. Cullin genes were not found in bacteria or archaea, but at least two cullins were found in all of the eukaryotic genomes that we examined (Table 2). The observation of cullins in protists suggests that the cullin gene family arose early in the eukaryotic lineage (Table 2, Fig. 2). All eukaryotic species examined contain cullins that were most similar to metazoan CUL1 and CUL4 in reciprocal BLAST analysis, with the exception of budding yeast (which lacks a CUL4-like gene) (Table 2). This suggests that an ancestral duplication that gave rise to CUL1-like and CUL4-like genes occurred early in eukaryotic evolution. This result matches a phylogenetic analysis of cullins, in which the first branch point of the cullin phylogeny creates two clades, with the first clade giving rise to CUL1, CUL2 and CUL5, and the second clade giving rise to CUL3 and CUL4 [75]. The adaptor proteins Skp1 and DDB1 are present whenever CUL1-like and CUL4-like genes are observed, suggesting that the association between the cullins and their adaptor proteins is ancient (Table 2, Fig. 2).

**Table 2:** Conservation of CUL4-DDB1^CDT2 ^and SCF^Skp2 ^components in prokaryotic and eukaryotic species.

**Group**	**Phylum or Division**	**Species**	Cullins*	**DDB1**	**CDT2**	**Skp1**	**Skp2**
**Eubacteria**	Aquificae	*Aquifex aeolicus*	**-**	**-**	**-**	**-**	**-**
	Firmicutes	*Bacillus subtilis*	**-**	**-**	**-**	**-**	**-**
	Proteobacteria	*Escherichia coli*	**-**	**-**	**-**	**-**	**-**
**Archaea**	Crenarchaeota	*Aeropyrum pernix*	**-**	**-**	**-**	**-**	**-**
	Euryarchaeota	*Methanocaldococcus jannaschii*	**-**	**-**	**-**	**-**	**-**
**Protist**	Apicomplexa	*Plasmodium falciparum*	2 (1, 4)		**-**		**-**
	Euglenozoa	*Leishmania infantum*	7 (1, 1, 3, 3, 3, 4, 4)		**-**		**-**
**Slime Mold**	Amoebozoa	*Dictyostelium discoideum*	5 (1, 1, 1, 3, 4)		**-**		**-**
**Plant**	Magnoliophyta	*Arabidopsis thaliana*	9 (1, 3, 3, 3, 4, 4, 4, 4, 4)				**-**
	Magnoliophyta	*Oryza sativa*	9 (1, 3, 3, 3, 3, 4, 4, 4, 4)				**-**
**Fungi**	Ascomycota	*Saccharomyces cerevisiae*	3 (1, 2, 3)	**-**	**-**		**-**
	Ascomycota	*Schizosaccharomyces pombe*	3 (1, 3, 4)				**-**
	Basidiomycota	*Cryptococcus neoformans*	7 (1, 1, 1, 1, 3, 3, 4)				**-**
**Invertebrate**	Nematoda	*Caenorhabditis elegans*	6 (1, 1, 2, 3, 4, 5)				
	Arthropoda	*Drosophila melanogaster*	5 (1, 1, 2, 3, 4)				
**Vertebrate**	Chordata	*Danio rerio*	8 (1, 1, 1, 2, 3, 3, 4, 4)**				
	Chordata	*Xenopus laevis*	6 (1, 1, 2, 3, 3, 4)				
**Mammalian**	Chordata	*Mus musculus*	6 (1, 2, 3, 4, 4, 5)				
	Chordata	*Bos taurus*	6 (1, 2, 3, 4, 4, 5)				
	Chordata	*Homo sapiens*	7 (1, 2, 3, 4, 4, 5, 7)				

A recognizable homolog in the species is designated by a check mark, and the absence of a homolog by a dash.

* The number of cullins is recorded in each organism. The metazoan cullin (CUL1-CUL5) for which each cullin is most related (by reciprocal BLAST analysis) is indicated in parentheses (e.g., 2 (1, 4) = two cullins that are most related to CUL1 and CUL4, respectively). The divergent CUL7 is listed separately for humans.

**For *Danio rerio *(zebrafish), genes that were predicted to encode cullin proteins of less than 100 amino acids were not included.

The substrate-specific components CDT2 and Skp2 appear to have arisen at different points in eukaryotic evolution. CDT2 is observed in all animals analyzed, and a majority of fungi and plants, but is not observed in protists (Table 2, Fig. 2). This suggests that CDT2 arose in the main ancestral eukaryote lineage after the protist lineages diverged, but prior to the genesis of plants. In contrast, Skp2 apparently arose later in evolution. Skp2 is present in animals, but is not detected in fungi or plants, suggesting that it arose after the branching of fungi from the main eukaryotic lineage but prior to the genesis of metazoa (Table 2, Fig. 2). This analysis implies that CDT2, and by extension the CUL4-DDB1^CDT2 ^complex, is more ancient than Skp2 and the SCF^Skp2 ^complex.

The finding that the CUL4-DDB1^CDT2 ^complex targets Cdt1 for degradation in fission yeast and *C. elegans*, while SCF^Skp2 ^does not, suggests that the CUL4-DDB1^CDT2 ^pathway is the ancient, conserved pathway for controlling the extent of DNA replication via Cdt1 degradation. A prediction of this hypothesis is that yeast or metazoan species that have lost genes for the CUL4-DDB1^CDT2 ^complex would have to employ a different strategy to restrict Cdt1 activity during S phase. In this regard, it should be noted that budding yeast (unlike other fungi) does not contain CUL4, DDB1, or CDT2 (Table 2). Strikingly, budding yeast employ a strategy for regulating Cdt1 that has not been observed in any other species: Cdt1 is exported from the nucleus with the Mcm2-7 complex rather than being degraded [3]. The fungal ancestor of budding yeast must have originally had the genes for the CUL4-DDB1^CDT2 ^complex and then lost them, because the genes are found in plants and other fungi (Fig. 2). It is possible that the loss of these genes put pressure on budding yeast to develop a novel strategy to regulate Cdt1 during S phase. Alternatively, the nuclear-export strategy may have developed and co-existed with the CUL4-DDB1^CDT2 ^pathway, but the redundancy between the two pathways subsequently allowed the loss of the CUL4-DDB1^CDT2 ^genes.

## Conclusion

In humans, both CUL4-DDB1^CDT2 ^and SCF^Skp2 ^redundantly target Cdt1 for degradation. SCF^Skp2^-mediated degradation of Cdt1 is not restricted to S phase in humans, but instead occurs throughout the cell cycle. In contrast, CUL4-DDB1^CDT2^-mediated degradation of Cdt1 is S-phase specific. The current evidence suggests that in fission yeast, *C. elegans*, *Xenopus*, and potentially even in mice, SCF^Skp2 ^does not contribute significantly to Cdt1 regulation, while the CUL4-DDB1^CDT2 ^complex is a major regulator of Cdt1 degradation in these species. The extent to which SCF^Skp2^-mediated Cdt1 degradation is conserved in mammals other than humans is not yet clear. Genome comparisons suggest that the CUL4-DDB1^CDT2 ^complex arose earlier in evolution than SCF^Skp2 ^based on the finding that a CDT2 ortholog is present in plants and fungi, while a Skp2 homolog is absent in these organisms. We propose that CUL4-DDB1^CDT2 ^is the ancient and paramount ubiquitin ligase for the degradation of Cdt1 in response to S-phase entry and DNA damage. Further experiments will be required to address the interesting question of when during early eukaryotic evolution the CUL4-DDB1 complex first began to regulate DNA replication.

## Competing interests

The author(s) declare that they have no competing interests.
